# CSF synaptic biomarkers and cognitive impairment in multiple sclerosis

**DOI:** 10.1007/s00415-024-12851-x

**Published:** 2024-12-21

**Authors:** Lorenzo Barba, Lorenzo Gaetani, Silvia Sperandei, Elena Di Sabatino, Samir Abu-Rumeileh, Steffen Halbgebauer, Patrick Oeckl, Petra Steinacker, Lucilla Parnetti, Massimiliano Di FIlippo, Markus Otto

**Affiliations:** 1 Department of Neurology, Martin-Luther-University of Halle-Wittenberg, Ernst-Grube-Strasse 40, 06120 Halle (Saale), Germany; 2 Section of Neurology, Department of Medicine and Surgery, University of Perugia, Piazzale Gambuli 1, 06129 Perugia, Italy; 3 Department of Neurology, University of Ulm, Ulm, Germany; 4 German Center for Neurodegenerative Diseases (DZNE, e.V.), Ulm, Germany

**Keywords:** CSF, SNAP-25, Beta-synuclein, Neurogranin, MS, Cognitive impairment

## Abstract

**Background:**

People with multiple sclerosis (PwMS) experience various degrees of cognitive impairment (CI). Synaptic dysfunction may contribute to CI in PwMS but cerebrospinal fluid (CSF) synaptic biomarkers are unexplored in MS.

**Objective:**

To assess the role of CSF synaptosomal-associated protein 25 (SNAP-25), β-synuclein, neurogranin and neurofilament light chain protein (NfL) in patients with early relapsing MS with and without CI.

**Methods:**

We measured CSF SNAP-25, β-synuclein, and neurogranin in 48 untreated PwMS and 50 controls with other neurological diseases (ONDs) and tested their associations with neuropsychological and MRI data.

**Results:**

CSF synaptic protein levels did not discriminate between MS subjects and patients with ONDs, with only SNAP-25 values being slightly increased in MS (*p* = 0.009). CSF synaptic markers were positively correlated with each other and with CSF NfL. Moreover, lower biomarker levels were found to be correlated with longer disease duration and lower brain volumes (especially of the thalamus). Moreover, we found significantly lower CSF SNAP-25 (*p* = 0.025), β-synuclein (*p* = 0.044), and neurogranin (*p* = 0.007) levels in PwMS with vs. without domain-specific cognitive impairment.

**Conclusion:**

Lower CSF synaptic biomarker levels were found in PwMS with longer disease duration and lower brain volumes and may identify PwMS at risk of CI.

**Supplementary Information:**

The online version contains supplementary material available at 10.1007/s00415-024-12851-x.

## Introduction

People with multiple sclerosis (PwMS) develop cognitive impairment (CI) in more than half of cases during the disease course, with neurocognitive symptoms ranging from mild cognitive dysfunction to dementia [1, 2]. The pathophysiology of CI in PwMS is complex and involves both focal and diffuse damage to white matter and grey matter structures, including the cortex, subcortical nuclei, and the cerebellum [3]. Synaptic damage and dysfunction are thought to play a key role in the pathogenesis of MS-related CI, as synaptic integrity is critical in brain networks underlying cognitive processes [3, 4]. Together with neurological examination, neuropsychological testing, and neuroimaging, fluid biomarkers reflecting different pathophysiological mechanisms may aid clinicians in the diagnostic and prognostic assessment of PwMS [5, 6]. In MS, currently used biomarkers in clinical practice mostly focus on immune pathways (e.g., B-cell activation markers) and neuroaxonal damage (neurofilament light chain protein, NfL) [7]. Instead, biomarkers reflecting synaptic dysfunction and/or damage (such as neurogranin, synaptosomal-associated protein 25, SNAP-25, and β-synuclein) have been investigated mostly investigated in cerebrospinal fluid (CSF) samples of patients with neurodegenerative diseases [8–12], but are unexplored in MS.

In this pilot study, we aimed to investigate a panel of CSF synaptic biomarkers, namely SNAP-25, neurogranin, and β-synuclein, in a well-characterized pilot cohort of untreated PwMS. We tested associations between synaptic markers, clinical and neuropsychological data, brain magnetic resonance imaging (MRI) as well as CSF NfL levels.

## Methods

### Case selection and clinical assessment

In this retrospective study, we analyzed a total of 98 CSF samples collected from 48 PwMS and 50 control subjects with other neurological diseases (ONDs) recruited at the Section of Neurology of the University of Perugia (Perugia, Italy). All PwMS had a diagnosis of relapsing MS formulated by trained neurologists according to the 2017 revision of the McDonald criteria [13] and met the following inclusion criteria: (i) CSF samples collected in the context of the routine diagnostic work-up, (ii) were never exposed to disease-modifying therapies and were steroid-free for at least 30 days before CSF sampling; (iii) no personal history of alcohol or drug abuse and of learning disability. As part of the routine diagnostic work-up, all patients underwent, at baseline, an extensive neurological examination, neuropsychological evaluation, brain MRI, and lumbar puncture. In all PwMS, the disease-related disability was assessed through the Expanded Disability Status Scale (EDSS) [14].

The control group included 50 subjects undergoing lumbar puncture during the diagnostic work-up in the suspicion of ONDs (*n* = 4 psychiatric symptoms, *n* = 9 polyneuropathy, *n* = 1 myasthenia gravis, *n* = 1 headache, *n* = 3 non-inflammatory optic neuropathy, *n* = 4 idiopathic intracranial hypertension, *n* = 5 functional disorders, *n* = 3 cerebrovascular diseases, *n* = 1 spinal cerebellar ataxia, *n* = 5 epilepsy, *n* = 13 subjective memory complaints with normal CSF Alzheimer’s disease biomarkers, i.e., A-T- profiles according to the 2018 NIA-AA Research Framework for a biological definition of Alzheimer’s disease) [15].

### Neuropsychological evaluation

Neuropsychological testing was carried out in all PwMS by a trained neuropsychologist within 60 days from CSF sampling. The Rao’s Brief Repeatable Battery of neuropsychological tests (BRB) were adopted to assess the domain-specific cognitive functioning, in particular: (i) verbal learning (VL) with the Selective Reminding Test (SRT), Long-Term Storage (SRT-LTS), Consistent Long-Term Retrieval (SRT-CLTR), and Delayed Recall (SRT-DR); (ii) visuospatial learning (VSL) with the Spatial Recall Test (SPART) and SPART Delayed Recall (SPART-DR); (iii) information processing speed (IPS) with the Paced Auditory Serial Addition Test (PASAT-3 and PASAT-2) and Symbol Digit Modalities Test (SDMT); (iv) verbal fluency (VF) on semantic input with the Word List Generation (WLG) test. The test scores were considered abnormal if lower than the 5th percentile relative to reference values for the Italian population adjusted according to sex and education [16]. PwMS were classified as having a domain-specific cognitive impairment (DSI) if they failed in at least one test exploring that domain (i.e., if the test score was at least 1.5 standard deviation below the normative reference values) [16]. Given the discrepancies between studies according to the criteria used to define CI in MS [17] and the exploratory nature of this study, we compared PwMS with at least one impaired cognitive domain vs. those with no impaired domains [18].

### MRI data acquisition and post-processing

Brain MRI examinations were performed in the context of the usual diagnostic work-up with a 1.5 T magnet (General Electric Medical Systems, Milwaukee, WI, USA) with a standard head-coil at the University Hospital of Perugia, Perugia (Italy). MRI protocols followed guidelines from the Italian Neurological and Neuroradiological societies for MRI use in MS [19]. Brain T2 lesions number (T2LN) and volume (T2LV) have been calculated by means of automated identification and filling of brain lesions implemented on the SInLAB platform (http://www.sienaimaging.it/). The platform automatically pre-processes NIfTI files and provides a lesion map using artificial intelligence methods [20]. The operator can then modify the map to correct any errors. Finally, the system generates a report to obtain total T2LN and T2LV and periventricular (PV), deep white matter (DWM), juxtacortical (JC) and infratentorial T2LN and T2LV. Brain volumes together with cortical grey matter (CGM), thalamus, and hippocampal volumes were calculated using SIENA-X 2.0 implemented on the same platform.

### CSF samples analysis

CSF samples were collected at the University of Perugia (Perugia, Italy) according to standardized international guidelines [21] and aliquots were stored at −80° until analysis, which was performed at the Martin-Luther University of Halle-Wittenberg (Halle, Germany). CSF NfL level was measured with commercially available kits for the Ella microfluidic system (BioTechne, Minneapolis, USA) and CSF SNAP-25 was quantified using the Simoa SNAP-25 advantage kit on a HD-X platform (Quanterix, Billerica, USA). CSF β-synuclein concentrations were measured with an in-house established immunoassay, as previously described [22]. For all measurements, coefficients of intra- and inter-assay variability were < 10% and < 15%, respectively.

### Statistical analysis

Statistical analysis was performed with GraphPad v.8 (GraphPad Software, La Jolla, USA) and R studio v.4.2.2 (R Foundation, Vienna, Austria). Comparisons of continuous and categorical variables between two groups were performed by the Mann–Whitney *U* and *χ*^2^ tests, respectively. Correlations between continuous variables were computed with the Spearman’s coefficient. Associations were tested with univariable and multivariable logistic regression models. For multiple testing, we applied Bonferroni’s post hoc correction according to the number of total hypotheses for the correlations of CSF markers with, respectively, clinical variables (i.e., age, disease duration, EDSS), with MRI volumes, with neuropsychological scores, and with each other. A *p* value < 0.05 was considered for all analyses as the first level of statistical significance.

### Study protocol approval

The protocol of this study was approved by the local Ethics Committee of the University of Perugia (CER Umbria, approval numbers: 1287/08, 3933/21, 3944/21), and all participants gave written informed consent to research. The study was conducted in accordance with the ethical standards of the 1964 Helsinki Declaration and its recent modifications.

## Results

### Cohort description

Our cohort included 50 subjects with ONDs [mean age: 49.5 (sd: ± 15.8) years, 60.0% females] and 48 PwMS [mean age: 37.3 (± sd: 9.8) years, 68.8% females] (Table 1). We found no significant differences in sex distribution between groups, whereas people with ONDs were significantly older than PwMS (*p* < 0.001). Hence, biomarker comparisons were tested also after accounting for age. In MS, median disease duration (DD) from symptom onset to lumbar puncture was 2.5 months (interquartile range, IQR: 1–12) and median EDSS score was 1.5 (IQR: 1–2). All PwMS were untreated at time of recruitment. PwMS had significantly higher cell count in CSF (*p* < 0.001) and higher IgG index [i.e., (CSF IgG / serum IgG) / (CSF albumin / serum albumin) or Q_IgG_ / Q_Alb_] (p < 0.001) compared to controls. Instead, the two groups did not significantly differ in blood parameters such as neutrophil count, lymphocytic count and neutrophil-to-lymphocyte ratio (NLR) (complete data in Table 1).

**Table 1:** Cohort demographics

	ONDs (*n* = 50)	MS (*n* = 48)	*p* value
Age	49.5 (± 15.8)	37.3 (± 9.8)	** < 0.001**
Male/female sex [n (%)]	20 (40.0) / 30 (60.0)	15 (31.2) / 33 (68.8)	0.488
Disease duration (m)	–	2.5 (1–12)	–
EDSS	–	1.5 (1–2)	–
Blood parameters			
Neutrophil count (cells/μl)	4396 (3539–5333)	3998 (2888–5698)	0.391
Neutrophils%	60.3 (52.8–68.0)	56.5 (47.9–64.8)	0.271
Lymphocyte count (cells/μl)	2167 (1691–2581)	2294 (1684–2668)	0.476
Lymphocytes%	31.2 (23.4–37.5)	32.7 (23.6–42.9)	0.236
Neutrophil-to-lymphocyte ratio (NLR)	1.93 (1.41–2.91)	1.73 (1.11–2.69)	0.258
CSF analysis			
Cell count (cells/μl)	0 (0–2)	2 (0–6)	**0.002**
Pleocytosis (%)	0 (0)	15 (31.3)	** < 0.001**
Positive OCB (%)	0 (0)	41 (85.4)	** < 0.001**
IgG index (Q_IgG_ / Q_Alb_)	0.49 (0.44–0.55)	0.66 (0.53–0.99)	** < 0.001**
CSF NfL (pg/ml)	570 (421–717)	746.5 (439.0–1447.2)	0.033 / 0.014*
CSF SNAP-25 (pg/ml)	57.1 (45–69)	63.5 (50.2–82.2)	0.039 / 0.009*
CSF β-synuclein (pg/ml)	169.9 (131.2–230.6)	172.7 (132.6–239.8)	0.843 / 0.360*
CSF neurogranin (pg/ml)	269.5 (189.0–374.5)	224.5 (161.2–294.5)	0.165 / 0.165*

Age is reported as mean (± sd), whereas other continuous variables as median (interquartile range, IQR). In bold significant* p* values < 0.05

*Given the age difference between groups, biomarker comparisons are reported also with age-adjusted *p* values

### Associations between CSF synaptic markers and clinical and biochemical parameters

**Fig. 1:**
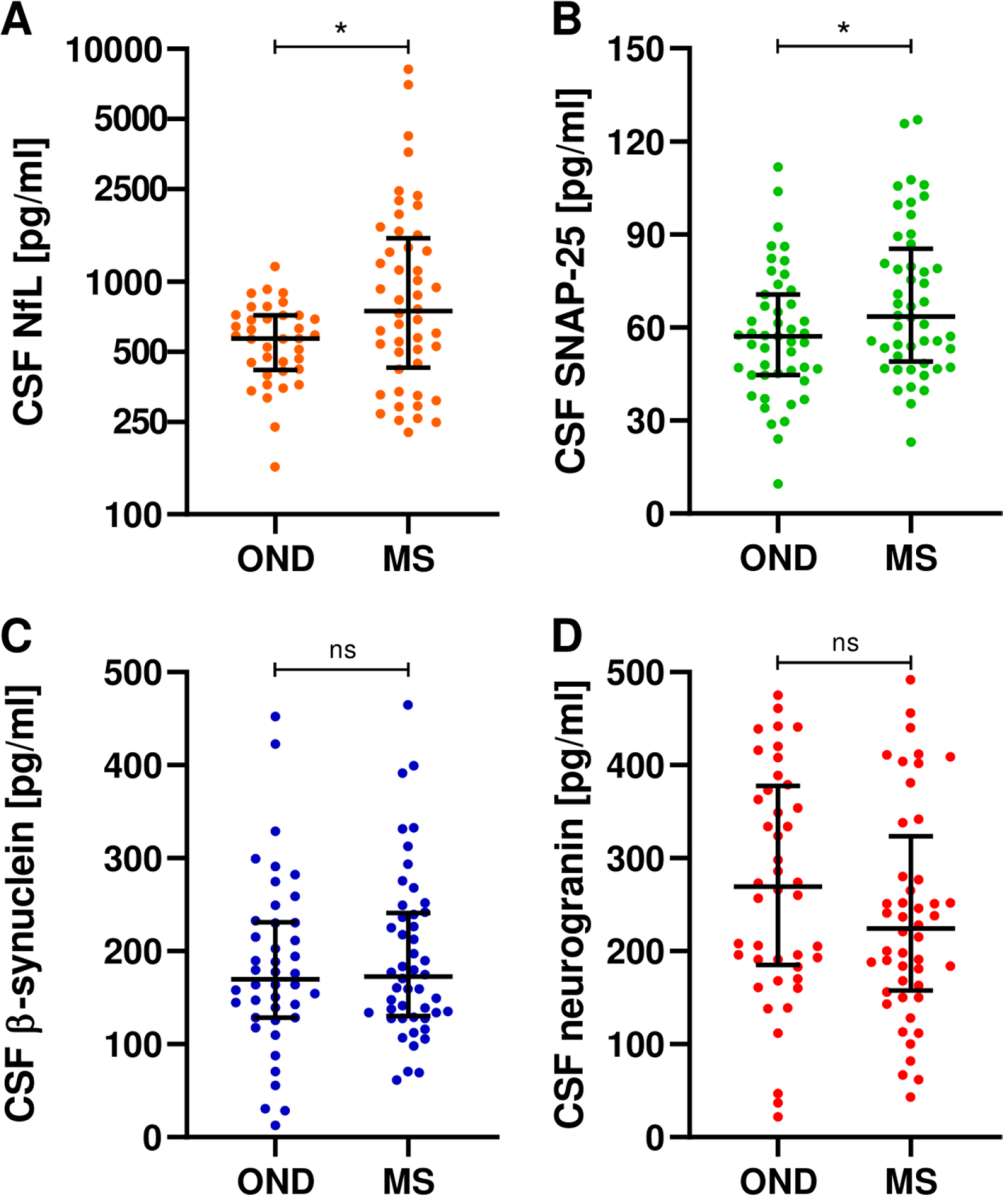
CSF biomarkers in people with multiple sclerosis (MS) and other neurological diseases (OND)

In our cohort, we found significantly increased CSF levels of NfL (*p* = 0.033, age-adjusted *p* = 0.014) and SNAP-25 (*p* = 0.039, age-adjusted *p* = 0.009) in MS compared to the OND group. Instead, CSF β-synuclein and neurogranin concentrations did not significantly differ between PwMS and subjects with ONDs (Fig. 1, Table 1).

**Fig. 2:**
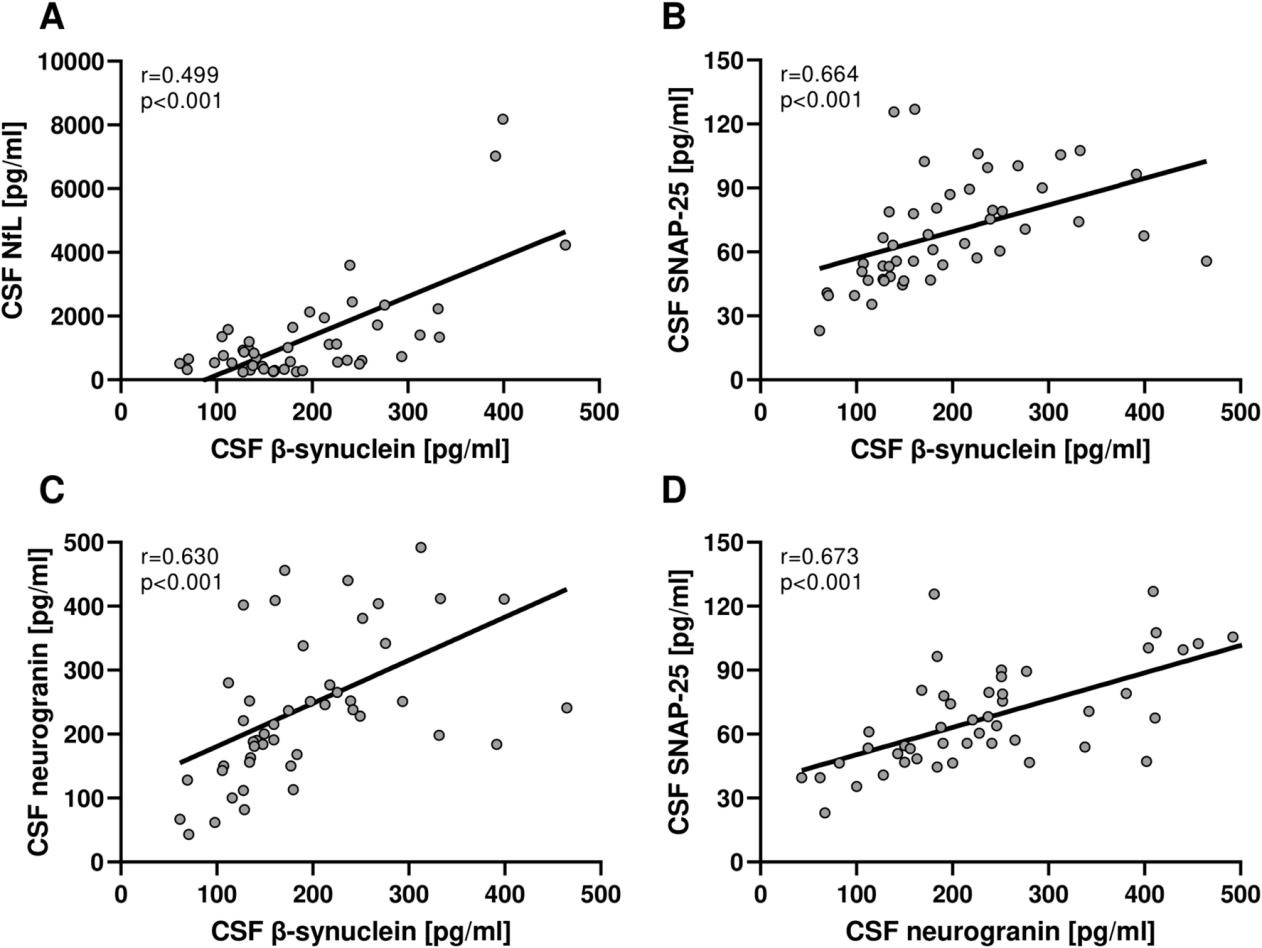
Spearman correlations between CSF biomarkers

In MS, age was not significantly correlated with CSF synaptic biomarker levels (Table 2). Interestingly, we found that CSF synaptic markers were well correlated with each other in PwMS (β-synuclein vs. SNAP-25 rho = 0.664, *p* < 0.001; β-synuclein vs. neurogranin rho = 0.630, *p* < 0.001; SNAP-25 vs. neurogranin rho = 0.673, *p* < 0.001) (Fig. 2, Table 2). Moreover, we found moderate correlations between β-synuclein and NfL (rho = 0.499, *p* < 0.001) and a trend toward a significant correlation between NfL and SNAP-25 (rho = 0.280, *p* = 0.054). Similar results were observed in the control group (Supplementary Tables S1). CSF synaptic marker levels were not significantly different in PwMS with (*n* = 41) vs. without (*n* = 7) CSF IgG oligoclonal bands. Interestingly, we found slightly increased CSF neurogranin levels (*p* = 0.046) and a trend toward more elevated CSF β-synuclein concentrations (*p* = 0.069) in PwMS with vs. without pleocytosis (i.e., CSF cell count > 4 cells/μl). At correlation analysis, CSF synaptic biomarkers were not significantly correlated with blood parameters (i.e., neutrophil count, lymphocytic count and NLR) nor with the IgG index (Table 2). We found a positive correlation between CSF β-synuclein concentrations and a higher CSF cell count (rho = 0.310, *p* = 0.034) but did not maintain statistical significance at Bonferroni’s correction (Table 2).

**Table 2:** Correlations between CSF markers in MS

	CSF NfL	CSF SNAP-25	CSF β-synuclein	CSF neurogranin
Age	ns	ns	ns	ns
DD in months	ns	rho = −0.411 *p* = 0.004*	ns	rho = −0.367 *p* = 0.010*
EDSS	ns	ns	ns	ns
Neutrophil count (cells/μl)	ns	ns	ns	ns
Neutrophils%	ns	ns	ns	ns
Lymphocyte count (cells/μl)	ns	ns	ns	ns
Lymphocytes%	ns	ns	ns	ns
Neutrophil-to-lymphocyte ratio (NLR)	ns	ns	ns	ns
CSF cell count	ns	ns	rho = 0.310 *p* = 0.034*	ns
IgG index (Q_IgG_ / Q_Alb_)	ns	ns	ns	ns
CSF NfL	–	ns	rho = 0.499 *p* < 0.001	ns
CSF SNAP-25	–	**–**	rho = 0.664 *p* < 0.001	rho = 0.673 *p* < 0.001
CSF β-synuclein	rho = 0.499 *p* < 0.001	rho = 0.664 *p* < 0.001	–	rho = 0.630 *p* < 0.001
CSF neurogranin	ns	rho = 0.673 *p* < 0.001	rho = 0.630 p < 0.001	–

^*^Reported *p* values did not maintain statistical significance after Bonferroni’s correction by adjusting for the number of hypotheses in the correlations between CSF markers and clinical variables (i.e., age, disease duration, EDSS) and with each other

CSF levels of SNAP-25 and neurogranin were negatively correlated with the disease duration in MS (rho = −0.411, *p* = 0.004 and rho = −0.367, *p* = 0.010, respectively). Statistical significance of these correlations was not maintained at Bonferroni’s post hoc correction. CSF synaptic biomarker levels were not significantly associated with the EDSS score (Table 2). PwMS with last relapse over 60 days before from CSF sampling had significantly lower levels of CSF neurogranin (*p* = 0.035) and SNAP-25 (*p* = 0.014) compared to subjects with recent relapse within 60 days. When considering relapses within 30 days from lumbar puncture, we found significantly higher CSF levels of SNAP-25 in PwMS with a recent relapse compared to other PwMS (*p* = 0.017, age-adjusted *p* = 0.010) and to controls (*p* = 0.003, age-adjusted *p* = 0.001). We found similar results for CSF NfL (*p* = 0.027, age-adjusted *p* = 0.025 vs. controls); instead, CSF β-synuclein and neurogranin did not significantly differ according to relapse within 30 days (Supplementary Fig. S1).

### Associations between CSF markers and MRI data

**Fig. 3:**
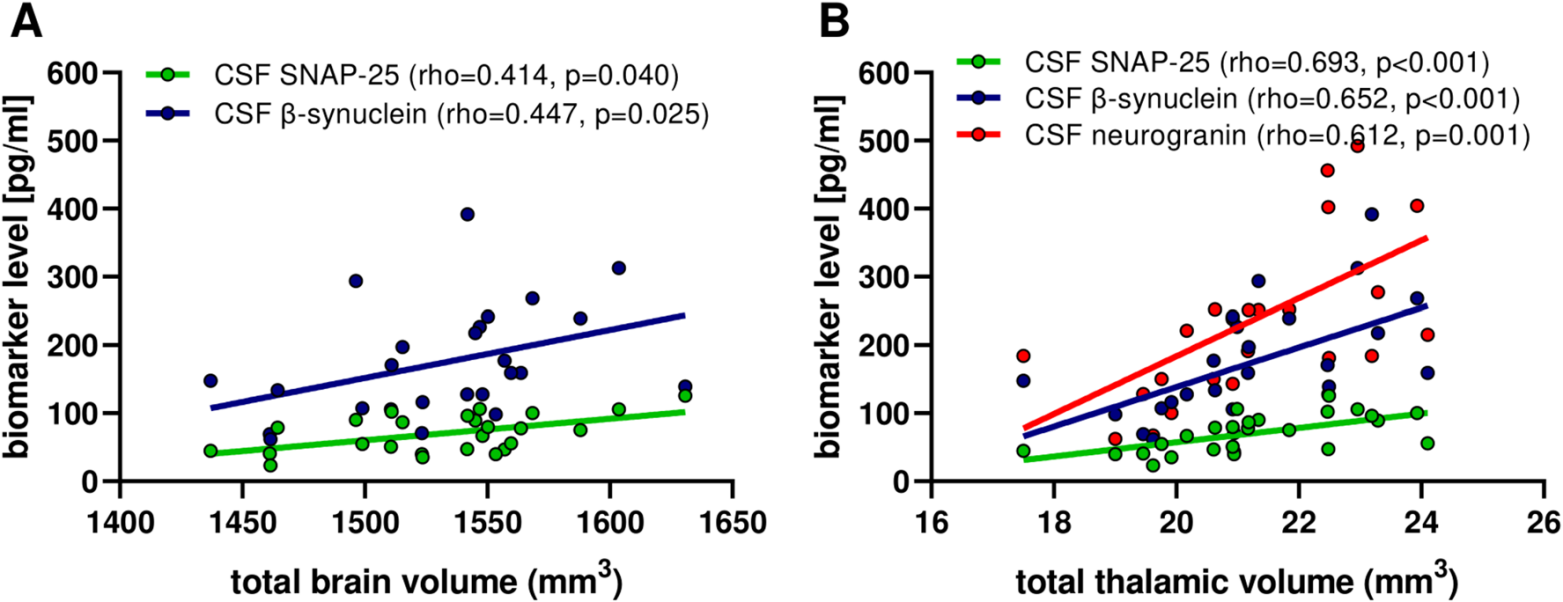
Correlations between CSF synaptic biomarkers and brain volumes at MRI

In PwMS with completely available MRI data (*n* = 25), we observed that both CSF SNAP-25 (rho = 0.414, *p* = 0.040) and β-synuclein (rho = 0.447, *p* = 0.025) were positively correlated with the total brain volume. Interestingly, correlations were even stronger between CSF synaptic markers and total thalamic volumes (rho = 0.652, *p* < 0.001 for β-synuclein; rho = 0.693, *p* < 0.001 for SNAP-25; rho = 0.612, *p* = 0.001 for neurogranin) (Fig. 3**, **Table 3). After accounting for age and disease duration in multivariable regression models, associations between lower CSF synaptic markers and lower thalamic volumes maintained statistical significance (Supplementary Table S2). In our cohort, CSF synaptic marker levels did not differ between PwMS with vs. without Gd-enhancing lesions at MRI (Supplementary Table S3). Moreover, we did not find significant correlations between CSF synaptic markers and lesions number/volume at T1- and T2-weighted MRI.

**Table 3:** Correlations between CSF markers and total volumes observed at MRI in MS. Adjusted *p* values were obtained at multivariable regression analysis after accounting for age and disease duration

	CSF NfL	CSF SNAP-25	CSF β-synuclein	CSF neurogranin
Whole brain	–	rho = 0.414 *p* = 0.040*	rho = 0.447 *p* = 0.025*	–
Cortical gray matter	–	–	–	–
Thalamus	–	rho = 0.693 *p* < 0.001	rho = 0.652 *p* < 0.001	rho = 0.612 *p* = 0.001
Hippocampus	–	–	–	–

^*^Reported *p* values did not maintain statistical significance after Bonferroni’s correction by adjusting for the number of hypotheses in the correlations CSF markers with MRI volumes

### Associations between CSF markers and neuropsychological data

In our cohort, CSF synaptic markers were not significantly correlated with the individual neuropsychological test scores from Rao’s BRB in PwMS. Moreover, they did not significantly correlate with the number of impaired tests or impaired cognitive domains.

**Fig. 4:**
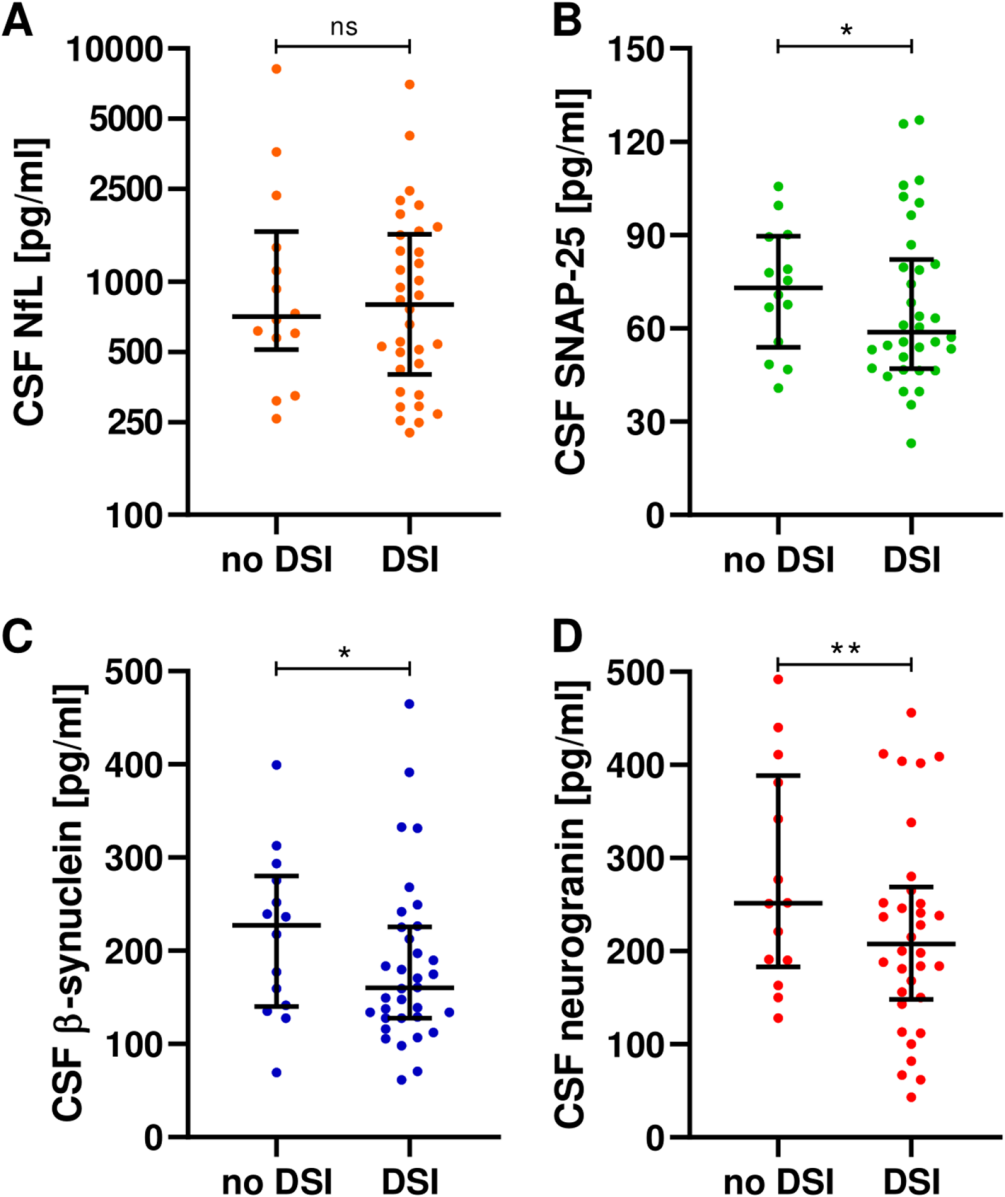
CSF synaptic biomarkers in people with multiple sclerosis with and without domain-specific cognitive impairment (DSI)

By comparing people with MS with vs. without DSI [18], we found significantly decreased CSF synaptic markers levels in the first compared to the latter group (β-synuclein *p* = 0.044; SNAP-25 *p* = 0.025; neurogranin *p* = 0.007) (Fig. 4, Supplementary Table S4). Low SNAP-25 and neurogranin levels were still associated with DSI after accounting for age, disease duration, EDSS, and Gd-enhancing lesions [SNAP-25 OR: 0.966 (95% confidence interval, 95%CI: 0.937–0.997), *p* = 0.029; neurogranin OR: 0.991 (95%CI: 0.985–0.998), *p* = 0.009] (Supplementary Table S5).

## Discussion

In this pilot study, we investigated for the first time a panel of CSF synaptic proteins in a cohort of drug-naïve PwMS. Interestingly, decreased CSF concentrations of SNAP-25, neurogranin and β-synuclein were found with patients with longer disease duration and lower brain volumes as well as they were associated with a higher chance of having DSI. Furthermore, CSF SNAP-25, but not neurogranin and β-synuclein concentrations, were significantly increased in PwMS with a relapse within 30 days compared to controls and the other PwMS. Our results suggest that PwMS with different clinical features, radiological characteristics, and disease course may experience various degrees of synaptic impairment/dysfunction, which could be associated with the individual risk of developing CI. However, even if very intriguing, the interpretation of such findings remains, to date, mainly speculative given the limited literature on synaptic markers in MS. Indeed, most of the data on SNAP-25, β-synuclein, and neurogranin in CSF derive from studies on neurodegenerative disease, such as Alzheimer’s, prion, and Lewy body diseases [8–12]. Here, the running hypothesis is that the ongoing neurodegeneration may lead to protein release from damaged synapses, which results in increased synaptic protein concentrations in CSF and then in peripheral blood [23, 24]. On the other side, the decrease of CSF levels of proteins reflecting synaptic pathway integrity, such as neuronal pentraxins, VGF, and neuroserpin, was associated with cognitive impairment in neurodegenerative diseases [25, 26]. Hence, we can speculate that lower CSF synaptic marker concentrations may reflect synaptic impairment leading to cognitive decline. However, if this hypothesis may also apply to MS is unclear, given that only one study in literature has explored synaptic protein levels in CSF of patients with inflammatory CNS disorders, i.e., antibody-mediated encephalitis (AME) [27]. Here, CSF concentrations of SNAP-25 and neurogranin were found to be decreased in patients with AME compared to controls as a possible marker of synaptic depression/dysfunction, but higher protein levels were associated with an overall more aggressive disease course [27]. As a possible explanation, the impairment of the synaptic function may be due to antibody-mediated internalization of proteins expressed in the neuronal/synaptic surface [28]. In MS, it has been hypothesized that synaptic dysfunction may underlie CI both as direct immune-mediated CGM involvement and as a consequence of disrupted cortico-subcortical networks, especially in the thalamus (e.g., “disconnection syndrome”) [3]. In agreement with this, we found correlations between CSF synaptic markers and both DSI and reduced brain volume, especially in the thalamus.

On another issue, we provided the first data in PwMS concerning CSF levels of β-synuclein, which was demonstrated to be potentially involved in MS pathogenesis [29]. Indeed, T lymphocytes reactive against β-synuclein were isolated in patients with MS, especially in progressive forms, as possible mediators of chronic grey matter damage [29]. Here, the fact is that β-synuclein CSF concentrations were reduced in PwMS with DSI and lower brain and thalamic volumes may hypothetically reflect autoimmune-mediated CGM involvement. However, the possible influence of T-cell- and/or antibody-mediate autoimmunity against cortical antigens on fluid synaptic protein levels in MS is still completely unexplored.

When testing the association between synaptic markers and a robust marker of axonal damage, such as NfL, we found only moderate positive correlations, especially for β-synuclein. This underscores the peculiar characteristics of NfL and synaptic markers, which may serve as complementary markers reflecting distinct topographic burdens of neuronal injury. Interestingly, CSF NfL was shown to be a valid indicator of acute axonal injury linked to focal Gd-enhancing lesions and to the overall visible lesional load on T2- and T1-weighted MRI [30, 31], whereas CSF synaptic proteins did not. These results suggest that, in MS, macroscopic focal white matter lesional load causing brain networks disconnection might be better reflected by an increase in axonal damage markers such as NfL rather than by synaptic markers. The latter, instead, could reflect the overall loss of synaptic structure and/or functionality accompanying MS along with the evolution of brain atrophy, contributing to the brain network failure underlying MS-related CI [32].

The main limitation of this study is the small sample size and the incomplete data concerning MRI. This hampers the generalizability of our results, which require external validation in independent and larger cohorts. Second, we lacked data on disease course and evolution at follow-up and, similarly to a previous study on AME [27], available data on synaptic markers are limited to people with disease duration of few months. This does not allow conclusions about the predictive value of such markers for future CI. On the other hand, we focused in this pilot study on a cohort of drug-naïve PwMS recruited at the time of the diagnosis. This allowed us to provide novel data very early in the disease course and without the possible confounding factor of pharmacological treatment. We included patients who had reported no corticosteroid exposure within 30 days prior to CSF sampling and who had never received disease-modifying therapies before. However, corticosteroid drugs may have effects persisting for more than 30 days, especially if administered systemically. Given that our cohort consists of otherwise healthy young adults, it is highly unlikely that they were exposed to corticosteroid before this period. Third, previous studies linked the CSF cytokine profile with the development and progression of CI in MS [33]. In particular, specific alteration patterns were found in PwMS experiencing mild and severe CI in comparison to cognitively unimpaired patients. Here, a CSF pro-inflammatory profile associated with elevated concentrations of B-cell related cytokines was associated with higher disease activity and more severe cortical damage [34], which may underlie synaptic dysfunction and ultimately CI. Even though we found no associations between CSF synaptic proteins and cell counts in peripheral blood, future studies will need to investigate better the relationship between CSF synaptic markers and CSF/blood cytokines and other markers of immunity. Also, they will need to include subjects with progressive disease course and under pharmacological treatment. Finally, pre-clinical studies on synaptic proteins will help to elucidate the pathophysiological mechanisms underlying the alterations of CSF synaptic protein levels in MS patients with and without CI.

In conclusion, our results show for the first time that CSF synaptic markers are associated with specific clinical and MRI characteristics of MS. A decrease in the tested proteins may occur with longer disease duration, lower brain volumes, and neuropsychological impairment. The role of CSF neurogranin, SNAP-25, and β-synuclein should be further investigated to better assess synaptic dysfunction and/or damage in MS.

## Supplementary Information

Below is the link to the electronic supplementary material.

Supplementary file1 (DOCX 19 KB)

Supplementary file2 (TIFF 327 KB)

## Data Availability

The anonymized data supporting the findings of this study are available from the corresponding author upon reasonable request.
