# Supplementary material for: CSF synaptic biomarkers and cognitive impairment in multiple sclerosis
Source: J Neurol. 2024 Dec 21;272(1):85. doi: 10.1007/s00415-024-12851-x (PMC11663154; doi:10.1007/s00415-024-12851-x)
Supplement: Supplementary file 1 — Supplementary file1 (DOCX 19 KB) [file 415_2024_12851_MOESM1_ESM.docx]

**Supplementary Table S1.** Correlations between CSF markers in control subjects.

|  | **Age** | **CSF NfL** | **CSF SNAP-25** | **CSF β-synuclein** | **CSF neurogranin** |
| --- | --- | --- | --- | --- | --- |
| **Age** | - | rho = 472  p = 0.003 | rho = 0.333  p = 0.022 | ns | ns |
| **CSF NfL** | rho = 472  p = 0.003 | - | rho = 0.408  p = 0.014* | rho = 0.378  p = 0.033* | ns |
| **CSF SNAP-25** | rho = 0.333  p = 0.022 | rho = 0.408  p = 0.014* | **-** | rho = 0.703  p < 0.001 | rho = 0.376  p = 0.013* |
| **CSF β-synuclein** | ns | rho = 0.378  p = 0.033* | rho = 0.703  p < 0.001 | - | rho = 0.495  p = 0.002 |
| **CSF neurogranin** | ns | ns | rho = 0.376  p = 0.013* | rho = 0.495  p = 0.002 | - |

*Reported p-values did not maintain statistical significance after Bonferroni’s correction by adjusting for the number of hypotheses in the correlations CSF markers with each other.

**Supplementary Table S2.** Multivariable regression models of the associations between CSF synaptic markers and thalamic volumes. Associations with other MRI volumes were not significant after adjustment for covariables.

| **Biomarker** | **Variables** | **β (95%CI)** | **t-value** | **p-value** |
| --- | --- | --- | --- | --- |
| CSF SNAP-25 | biomarker | 0.04 (0.02-0.06) | 3.33 | **0.003** |
|  | age | -0.01 (-0.06-0.05) | -0.24 | 0.814 |
|  | disease duration (months) | 0.002 (-0.01-0.01) | 0.24 | 0.811 |
| CSF β-synuclein | biomarker | 0.01 (0.004-0.02) | 2.93 | **0.008** |
|  | age | 0.001 (-0.06-0.06) | 0.03 | 0.979 |
|  | disease duration (months) | -0.002 (-0.01-0.01) | -0.38 | 0.705 |
| CSF neurogranin | biomarker | 0.006 (0.001-0.01) | 2.44 | **0.024** |
|  | age | 0.02 (-0.04-0.08) | 0.74 | 0.467 |
|  | disease duration (months) | -0.003 (-0.02-0.01) | -0.42 | 0.676 |

**Supplementary Table S3.** Demographic, clinical and biochemical characteristics of people with MS with vs. without Gd-enhancing lesions.

|  | **Gd- (n=24)** | **Gd+ (n=24)** | **p-value** |
| --- | --- | --- | --- |
| **Age** | 39.1 (±9.2) | 35.5 (±10.3) | 0.127 |
| **Male / female sex [n (%)]** | 8 (33.3) / 16 (66.7) | 7 (29.2) / 17 (70.8) | 0.999 |
| **Disease duration in months** | 3.5 (1-12) | 1.5 (1-12) | 0.851 |
| **EDSS** | 2 (1-2) | 1.25 (1-2) | 0.424 |
| **CSF NfL (pg/ml)** | 761 (334-1502) | 747 (484-1447) | 0.959 |
| **CSF SNAP-25 (pg/ml)** | 65.3 (49.9-79.9) | 62.1 (52.0-91.7) | 0.813 |
| **CSF β-synuclein (pg/ml)** | 175.9 (136.1-223.7) | 160.1 (132.6-241.7) | 0.869 |
| **CSF neurogranin (pg/ml)** | 238 (178-339) | 195 (155-269) | 0.613 |

**Supplementary Table S4.** Demographic, clinical and biochemical characteristics of people with MS with and without domain-specific cognitive impairment (DSI).

|  | **no DSI (n=17)** | **DSI (n=31)** | **p** |
| --- | --- | --- | --- |
| **Age** | 37.7 (±8.5) | 37.1 (±10.6) | 0.931 |
| **Male / female sex [n (%)]** | 8 (57.1) / 6 (42.1) | 4 (16.7) / 20 (83.3) | **0.006** |
| **disease duration (months)** | 1 (0.5-4) | 4 (1-12) | 0.087 |
| **EDSS** | 1.5 (1-2) | 2 (1-2) | 0.391 |
| **CSF NfL (pg/ml)** | 686 (551-1338) | 836 (433-1615) | 0.932 |
| **CSF SNAP-25 (pg/ml)** | 77.9 (66.7-99.5) | 55.7 (47.0-76.5) | **0.025** |
| **CSF β-synuclein (pg/ml)** | 226.5 (159.4-275.8) | 149.2 (127.5-205.0) | **0.044** |
| **CSF neurogranin (pg/ml)** | 277 (191-412) | 198 (147-249) | **0.007** |

**Supplementary Table S5.** Associations between CSF synaptic markers and (DSI) in MS.

| **Biomarker** | **Model** | **Variables** | **OR (95%CI)** | **z-value** | **p-value** |
| --- | --- | --- | --- | --- | --- |
| CSF SNAP-25 | Univariate | biomarker | 0.974 (0.949-1.0002) | -1.95 | 0.052 |
|  | Multivariate | biomarker | 0.966 (0.937-0.997) | -2.18 | **0.029** |
|  |  | age | 0.983 (0.911-1.061) | -0.43 | 0.666 |
|  |  | disease duration (months) | 1.009 (0.985-1.034) | 0.75 | 0.452 |
|  |  | EDSS | 2.636 (0.917-7.573) | 1.80 | 0.072 |
|  |  | Gd-enhancing lesions | 2.275 (0.565-9.159) | 1.16 | 0.247 |
| CSF β-synuclein | Univariate | biomarker | 0.994 (0.988-0.1.001) | -1.57 | 0.118 |
|  | Multivariate | biomarker | 0.994 (0.987-1.002) | -1.49 | 0.136 |
|  |  | age | 0.985 (0.918-1.058) | -0.40 | 0.687 |
|  |  | disease duration (months) | 1.011 (0.987-1.035) | 0.89 | 0.373 |
|  |  | EDSS | 2.014 (0.801-5.065) | 1.49 | 0.137 |
|  |  | Gd-enhancing lesions | 1.780 (0.483-6.560) | 0.87 | 0.386 |
| CSF neurogranin | Univariate | biomarker | 0.992 (0.986-0.998) | -2.71 | **0.007** |
|  | Multivariate | biomarker | 0.991 (0.985-0.998) | -2.63 | **0.009** |
|  |  | age | 0.967 (0.892-1.048) | -0.83 | 0.409 |
|  |  | disease duration (months) | 1.005 (0.982-1.029) | 0.44 | 0.658 |
|  |  | EDSS | 2.148 (0.764-6.039) | 1.45 | 0.147 |
|  |  | Gd-enhancing lesions | 1.858 (0.438-7.877) | 0.84 | 0.401 |
