# Supplementary figures and images for: CSF synaptic biomarkers and cognitive impairment in multiple sclerosis
Source: J Neurol. 2024 Dec 21;272(1):85. doi: 10.1007/s00415-024-12851-x (PMC11663154; doi:10.1007/s00415-024-12851-x)

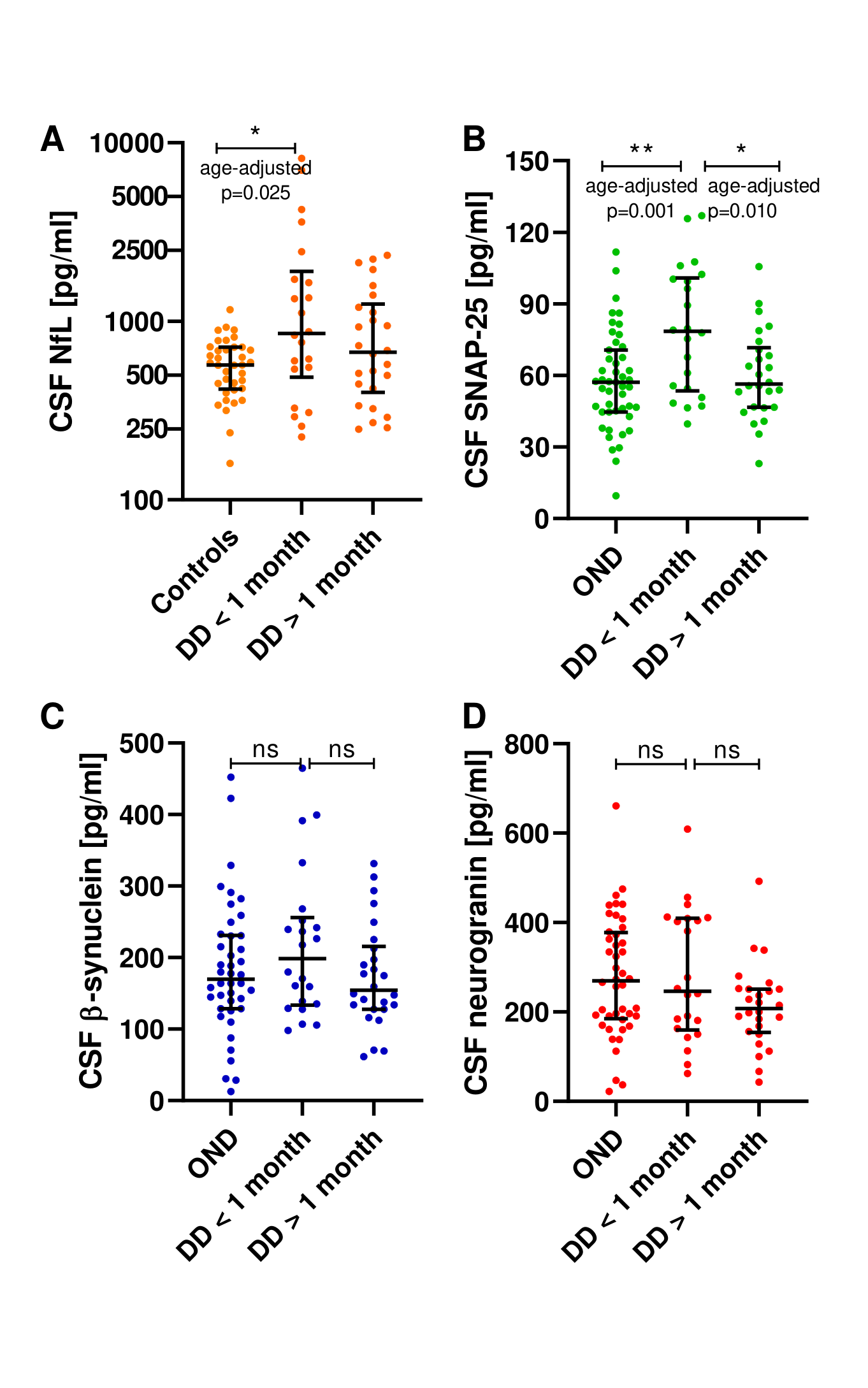

Supplement: Supplementary file 2 — Supplementary file2 (TIFF 327 KB) [file 415_2024_12851_MOESM2_ESM.tiff]
